# Unsupervised Dark-Channel Attention-Guided CycleGAN for Single-Image Dehazing

**DOI:** 10.3390/s20216000

**Published:** 2020-10-23

**Authors:** Jiahao Chen, Chong Wu, Hu Chen, Peng Cheng

**Affiliations:** 1 National Key Laboratory of Fundamental Science on Synthetic Vision, Chengdu 610000, China; chenjiahao@stu.scu.edu.cn (J.C.); chongwuscu@outlook.com (C.W.); scechina@163.com (P.C.); 2 School of Computer Science, Sichuan University, Chengdu 610000, China; 3 School of Aeronautics and Astronautics, Sichuan University, Chengdu 610000, China

**Keywords:** dehazing, GAN, attention, dark channel

## Abstract

In this paper, we propose a new unsupervised attention-based cycle generative adversarial network to solve the problem of single-image dehazing. The proposed method adds an attention mechanism that can dehaze different areas on the basis of the previous generative adversarial network (GAN) dehazing method. This mechanism not only avoids the need to change the haze-free area due to the overall style migration of traditional GANs, but also pays attention to the different degrees of haze concentrations that need to be changed, while retaining the details of the original image. To more accurately and quickly label the concentrations and areas of haze, we innovatively use training-enhanced dark channels as attention maps, combining the advantages of prior algorithms and deep learning. The proposed method does not require paired datasets, and it can adequately generate high-resolution images. Experiments demonstrate that our algorithm is superior to previous algorithms in various scenarios. The proposed algorithm can effectively process very hazy images, misty images, and haze-free images, which is of great significance for dehazing in complex scenes.

## 1. Introduction

In hazy weather, there are usually a large number of impurities (such as particulates and water droplets) in outdoor scenes. Due to the absorption and scattering of the atmosphere, haze, fog, and smoke are generated. The influence of these factors causes a certain degree of degradation in the image quality acquired by a camera, reducing the sharpness and contrast of the captured image. In severe cases, these factors can also distort the color information in the image, making computer vision technologies such as image recognition and image segmentation more difficult to apply. In recent years, with the popularization of computer vision systems and their application in various industries, these systems have played an important role in roads, aviation, and other fields. To enable these systems to function normally in various severe weather conditions, image clarity processing is essential. After the image acquisition device collects the hazy image, in order to facilitate the further operation of a program, image enhancement preprocessing operations are usually performed on the original image to improve its contrast, while rendering the target object clearer and easier to identify. For hazy images, this image enhancement operation is called image dehazing.

The dehazing operation for a single image has always been a key concern. Traditional methods are usually based on the parameter estimation of an atmospheric scattering model [1,2], represented by the dark channel, previously introduced by He et al. [3]; these methods mostly use prior knowledge for dehazing [3,4,5,6]. In recent years, deep learning has continuously developed, and many networks for image conversion have emerged. Through learning, network models can better transform images from the source domain to the target domain, where the effect of Goodfellow et al.’s [7] generation adversarial networks (GANs) is relatively good. GANs [7] use a confrontation between a generator and a discriminator to generate images with similar styles from the source domain to the target domain, and they have been widely applied in this field. CycleGAN [8] neither requires additional supervision nor paired datasets. Because the images are generated in a cyclic process between the two domains, the limitations of the dataset are greatly reduced, and good results can be obtained. The recently proposed CycleDehaze [9] uses an improved CycleGAN [8] to further enhance the texture and sharpness of an image by adding perceptual loss and to improve the effect of dehazing.

As algorithm synthesis makes it easier to create large-scale datasets that meet the needs of the network, most existing methods use artificially synthesized hazy images as the dataset. A synthetic hazy image is created according to an algorithm, and the distribution of the hazy areas has a certain regularity, which is very different from natural haze characteristics. The models and algorithms designed on the basis of this training have certain limitations in practical applications, and they cannot adequately remove natural haze. Therefore, this experiment uses the O-HAZE [10] dataset. This dataset contains 45 actual hazy images with a corresponding haze-free image for each case. The images have high spatial resolution, and each image was collected in a different scene with inconsistent haze concentrations, thereby appropriately reflecting actual dehazing situations.

In actual hazy images, the concentrations of haze in different regions and depths are inconsistent; using a simple method for overall image conversion results in uneven haze removal, with the resulting image having serious chromatic aberration. The existing overall image conversion methods do not pay attention to specific parts of an image, failing to focus on the area to be converted; thus, they are unable to retain the original features in mist- and haze-free areas. These algorithms may change areas that do not need to be processed, thereby leading to “false dehazing”.

**Figure 1:**
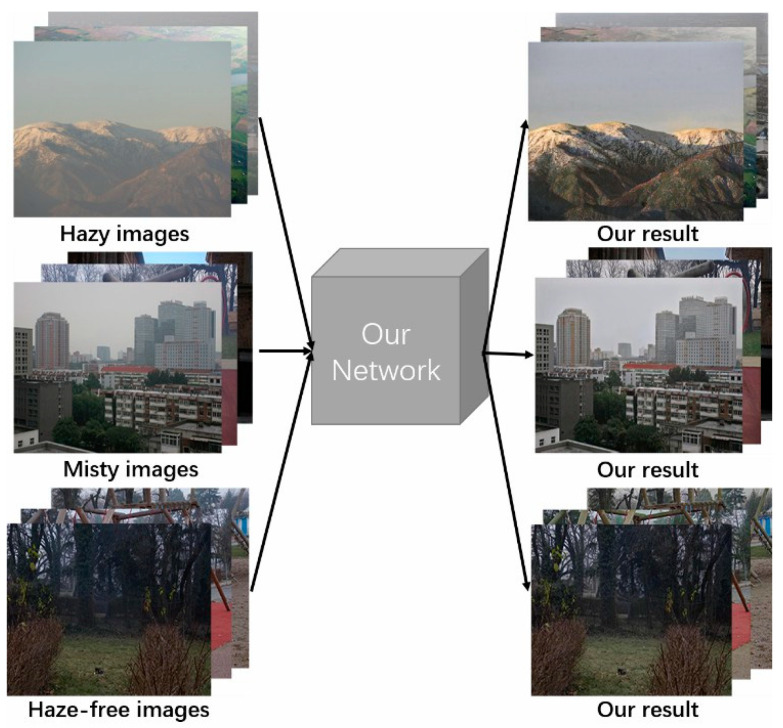
Our network can convert hazy images, misty images, and haze-free images into the results we expect.

In view of the above problems, this paper combines the classic dark-channel prior dehazing algorithm [3], noting that the algorithm has a similar starting point to the construction of attention maps in neural networks. The dark-channel map can better mark hazy areas in an image, and these areas are exactly those expected from the attention map. The pixel values of the dark-channel map also represent the concentration of haze, which is similar to an attention map. Therefore, the proposed method discards the attention network and calculates the dark-channel image obtained from the original image to be transformed, before sending the dark-channel image to the network as an attention map for training after a series of processes, such as normalization, coefficient enhancement, and guiding filtering. The experimental results show that the proposed method introduces a dark-channel-based attention mechanism into the network that not only reduces the cost of solving the attention map, but also achieves good results in actual complex dehazing situations. Figure 1 shows the results achieved in this study.

Our innovations can be summarized as follows:

1. Aiming at the characteristics of uneven concentrations of haze in different areas of an actual image, the proposed method enhances the network structure of CycleGAN [8], introduces a related attention mechanism, and completes an end-to-end partitioned component-dehazing process.
2. The proposed method innovatively uses the enhanced dark channel as the attention map and introduces the dark-channel enhancement coefficient. Through the training of the network, the enhanced dark-channel map can more effectively and accurately mark the hazy areas and their concentrations in an image, as well as increase the difference between different haze concentrations.
3. Benefiting from the characteristics of the dark channel and attention mechanism, the proposed network can better retain the original features and details of an image, and it is more robust to mist- and haze-free image conversion.

The subsequent chapters introduce work related to image dehazing. In Section 3, we describe in detail the implementation of our method. The experiments and results are presented and discussed in Section 4. The final chapter provides a summary and introduces potential future directions.

## 2. Related Work

Image dehazing has always been a popular research topic, especially as it relates to single-image dehazing. Traditional single-image dehazing methods are mostly based on image-dehazing theory, studied using prior information-based methods. Through many observations, He et al. [3] discovered the dark-channel principle. Using an atmospheric scattering model [1,2], a single-image dehazing transmission map can be calculated. The color-line method of Fattal [11] and the haze-line method of Berman et al. [12] were also introduced. Since prior information from humans is not always applicable to images of different contexts, these methods often cause image color distortion.

With the development of deep learning, convolutional neural networks (CNNs) have been successful in many computer vision tasks, and many single-image dehazing methods incorporating deep learning have emerged [13,14,15]. These methods used CNNs to design a model to estimate the transmission map, and then used an atmospheric scattering formula to recover the image. Ren et al. [16] designed a large-scale network to predict the overall transmission map, and then designed a suitable-scale network to redefine it. Cai et al. [17] developed an end-to-end dehazing network model called DehazeNet on the basis of a bipolar rectified linear unit (BReLU) feature-extraction layer. The transmission maps were predicted by convolutional neural networks, and then the atmospheric scattering model was used to remove haze; however, the inaccuracy in transmission-map estimation and the changes in atmospheric light were not considered, while the relationships among the internal transmission maps, atmospheric light, and dehazing results were ignored, which all reduced the overall dehazing effect. Therefore, Zhang et al. [2] proposed a separate dehazing network (DCPDN) to jointly learn the transmission map, atmospheric light, and haze-free images, thereby obtaining the relationships among them. Although the dehazing performance was improved, there were some limitations, as only the atmospheric scattering model was considered when designing the CNN.

With the rise of GANs [7], great progress has been made in the direct end-to-end generation of corresponding images [18,19]. Isola et al. [20] completed the style transformation of paired images through the conditional generation antagonism network (CGAN) [20]. GANs [7] are also often used in dehazing [21,22]. Visual Geometry Group Network (VGG) features was introduced by Li et al. [23,24] introduced, and the L1-regularized gradient of CGAN [20] was used for image dehazing. Isola et al. [20] used pix2pix to perform paired image translation. Engin et al. [9] used CycleGAN [8] to introduce perceptual loss and proposed an unsupervised dehazing network called CycleDehaze [9], which allows the network itself to learn the characteristics of hazy and haze-free images to perform end-to-end transformation, thereby resolving the relationship between haze-free and hazy features at a deeper level and further improving the overall dehazing effect of the image. However, the limitations of the overall style migration of the GANs [7] resulted in the model being unable to make smooth transitions in areas without haze or with little haze and being unable to achieve different degrees of conversion for areas with different haze concentrations. On this basis, we introduce the attention mechanism as a function of CycleDehaze [9], and we design an attention map which can obtain satisfactory dehazing effects in different situations.

## 3. Proposed Approach

The goal of image dehazing is to improve the clarity of the whole image; thus, past methods applied a global process to the image. For haze images synthesized by algorithms, the haze distribution and size are often regular; hence, the overall conversion method has a better dehazing effect. However, actual haze has characteristics of different image depths, different hazy areas, and uneven haze concentrations. A uniform conversion of the overall image is, thus, not particularly ideal, as it is impossible to distinguish between very hazy areas, misty areas, and haze-free areas. The proposed method implements the previously described CycleDehaze [9] network, enhances it, and applies it to solve the above-mentioned problem.

Attention mechanisms are often used to focus on specific objects or areas within an image. In our work, the transformed area is taken as the hazy area of an image, whereby the degree of transformation depends on the haze concentration. This is a local concern; therefore, it is necessary to introduce the attention mechanism into this dehazing approach.

**Figure 2:**
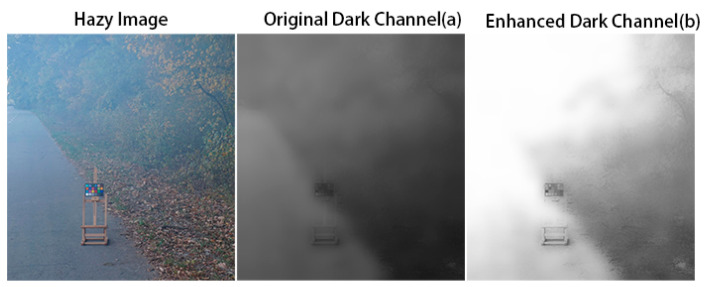
The original dark-channel map (**a**), and the dark-channel map after training and enhancement (**b**).

In view of the characteristics of actual hazy images, attention maps need to solve two main problems: (1) the labeling of hazy areas, and (2) the quantification of haze concentration. In response to the first problem, the proposed method uses dark channels to effectively reflect the hazy area. A larger value in a dark-channel map denotes greater haze concentration in the original image and a whiter area reflected in the attention map. However, the dark channel shown in Figure 2 is not ideal for quantifying the concentration. To increase the difference between haze concentrations, the proposed network introduces the coefficient *v* to enhance the attention map. To improve the adaptability of the attention map to network changes, the proposed method trains the coefficient *v* along with the network.

**Figure 3:**
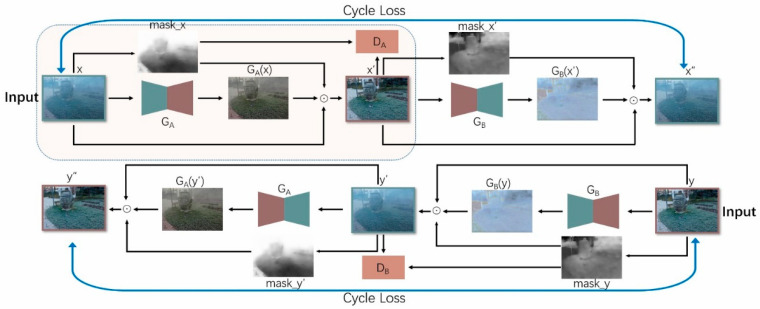
The structure of the proposed network and the intermediate process of image conversion.

The proposed method implements CycleDehaze [9] for generating dehazed images and introduces two generators named GA and GB, two discriminators named DA and DB, and the two attention enhancement coefficients named *v*. Similar to CycleDehaze [9], this network has a cyclic conversion process between hazy and haze-free images. In the conversion process from hazy to haze-free, a dark channel map is obtained after enhancement, guided filtering [25], and normalization, resulting in the attention map *mask_x* being obtained. Finally, GA(*x*), *x*, and *mask_x* are calculated using an elementwise product to obtain the final result. Figure 3 shows the overall structure of this network and the overall flow chart of the dehazing operation. In this figure, the GA generator is responsible for converting hazy images *x* and *y*′ into haze-free images *x*′ and *y*″, whereas the GB generator is responsible for converting haze-free images *x*′ and *y* into hazy images *x*″ and *y*′. DA and DB are the two discriminators, while ʘ represents the elementwise product operation. This figure also shows the cyclic generation process of the network, including the conversion between the target domain and the source domain; the upper-left shaded region describes the focus of our study through which we obtained results.

### 3.1. Attention Mechanism

The attention mechanism is an important part of this paper. It has been widely used in image conversion. Mejjati et al. [26] proposed using attention maps in GANs [7] to label objects that needed to be transformed, whereby attention networks were built and trained to reach areas where the objects of interest were located.

However, in image dehazing, the hazy area covers almost the whole image, while the level of haze is different; thus, the attention map obtained via training usually focuses on the sharp and prominent parts of the image. It is impossible to notice the actual hazy areas in the whole image, thereby making it impossible to accurately identify the haze concentration. Therefore, the dehazing effect is not ideal, as situations usually arise where the local effect is better, but the overall effect is biased, whereas some areas are not noticed.

The attention map in this paper was inspired by the dark-channel method of He et al. [3]. The dark-channel method proposed can accurately mark the location of haze, further characterizing it by normalizing its value to [0,1]. Compared with the method of Mejjati et al. [26], which requires more than 100 epochs for overall image conversion, haze concentration can greatly increase speed and accuracy. In reality, haze should be a continuous element of an image, with no obvious edges. Therefore, this article aimed to smooth the edges of objects affected by haze. In guidance filtering, a smoothing factor is appropriately increased to smoothen the obtained dark-channel map.

(1) $$J^{dark}\left(x\right)=\underset{y\in \Omega \left(x\right)}{\min}\left(\underset{c\epsilon \left\{R,G,B\right\}}{\min}J^{c}\left(y\right)\right).$$

Equation (1) describes the calculation method of the original dark-channel method [3], where Ω(x) is the area centered on x, $J^{c}$ is the color channel, and $J^{dark}$ is the dark channel of image J. As seen in Figure 2, the original dark channel can accurately display the haze area and concentration, but it is relatively gray; that is, the dark channel map has a lower value and cannot adequately represent the haze. The difference between the concentration and the reflection of different hazy areas may prevent dehazing from being targeted during the conversion process, thus enhancing the value of the dark-channel map.

(2) $$dark=\min\left\{v*J^{dark}\left(x\right),255\right\}.$$

Equation (2) allows enhancing the original dark channel to obtain a new dark channel. The proposed method applies a dark channel enhancement coefficient v for this purpose, with an initial value of 2. This coefficient is multiplied after the dark channel is generated, before adjusting and optimizing it with the training of the network to obtain a better coefficient, allowing a suitable attention map for our network to be generated.

### 3.2. Cyclic Generation Process

This network introduces cyclic consistency loss and perception loss. Similar to CycleGAN [8], images have a cyclic generation process in the network. The hazy image x is input into the network using the proposed method, thereby generating the image $G_{A}\left(x\right)$ through the first generator $G_{A}$. Next, the attention mechanism is applied using Equation (2) to simultaneously calculate mask_x.

The proposed method divides the final desired image into two parts; the first is the part of the original image that does not need to be converted, while the second is the part generated by the generator. The next step is to calculate and combine both parts.

For the final synthesis of the haze-free image, the proposed model uses the method of Mejjati et al. [26]. First, the elementwise product ʘ of mask_x and $G_{A}\left(x\right)$ is used to obtain the dehazing conversion part of the image (this step can be regarded as the image-dehazing operation of the haze area of the original image). Then, 1−mask_x and x are used to obtain the original part of the image, thereby retaining the haze-free area of the image. Finally, the two parts are combined to obtain the final haze-free image $x^{\prime }$. In this way, while dehazing the target area of the image, the clear parts and the details of the image that do not require conversion are also maintained. Equation (3) illustrates this process.

(3) $$x^{\prime }=mask_{x}ʘG_{A}\left(x\right)+\left(1-mask_{x}\right)ʘx,$$

where ʘ represents the elementwise product operation. To calculate the cyclic consistency loss and perception loss, the proposed method needs to perform another round of transformation on the obtained haze-free image $x^{\prime }$. Similarly, the attention map is determined using Equation (2), whereby $x^{\prime \prime }$ is generated by $G_{B}$. Due to the cyclic mechanism of CycleGAN [8], the conversion of haze-free image y to hazy image $y^{\prime \prime }$ was consistent with the above-described process.

### 3.3. Loss Function

To retain details in the image, the proposed method introduces the VGG16 [24] model to the network. This model is pretrained by ImageNet [27].

(4) $$L_{Perceptual}^{x}\left(x,x^{\prime \prime }\right)=∥vgg\left(x\right)-vgg{\left(x^{\prime \prime }\right)∥}_{2}^{2}.$$

The proposed method characterizes the values of *x*, *y*, *x*″, and *y*″ in Equation (3), using the VGG16 [24] model to extract the features of the 2_2nd convolutional layer and 5_3rd convolutional layer; this process is represented by “vgg()”. Then, proposed method uses the extracted features to calculate the cyclic perception loss [9], as shown in Equation (4).

Our final loss function ultimately includes three parts: the adversarial loss of the GANs [7], the cyclic consistency loss, and the perceptual loss [8,9]. Due to the cyclic structure of the network, it contains a total of six losses.

(5) $$L_{adv}^{x}\left(G_{A},A_{x},D_{A}\right)=E_{y\sim P_{Y}\left(y\right)}\left[\log\left(D_{A}\left(y\right)\right)\right]+E_{x\sim P_{S}\left(x\right)}\left[\log\left(1-D_{A}\left(x^{\prime }\right)\right)\right].$$

Equation (5) describes the adversarial loss of the GANs [7]. We set the adversarial loss for both the generator and the discriminator. When the generator establishes a fake haze-free image $x^{\prime }$, the discriminator also tries to distinguish it from actual haze-free images. Therefore, $G_{A}$ attempts to generate images as accurately as possible, whereas $D_{A}$ tries to distinguish the generated image $x^{\prime }$ from the actual haze-free image *y*.

(6) $$L_{cyc}^{x}\left(x,x^{\prime \prime }\right)=∥x-x^{\prime \prime }{∥}_{1}.$$

Equation (6) describes the cyclic consistency loss on the basis of CycleGAN [8]. In the network, the learned mapping function should have cyclic consistency. If the image is converted from one domain to another and then converted back, it should return to the original domain. Thus, we can subtract the values of x and $x^{\prime \prime }$ generated by $G_{A}$ and $G_{B}$ to obtain the cyclic consistency loss.

(7) $$L\left(G_{A},G_{B},A_{x},A_{y},D_{S},D_{T}\right)=L_{adv}^{x}+L_{adv}^{y}+\alpha_{cyc}\left(L_{cyc}^{x}+L_{cyc}^{y}\right)+\;\beta_{Perceptual}\left(L_{Perceptual}^{x}+L_{cyc}^{y}\right).$$

Equation (7) describes the calculation of all losses, where *A_x_* and *A_y_* represent two attention mechanisms.

(8) $$G_{x}^{*},G_{y}^{*},A_{x}^{*},A_{y,}^{*}D_{x}^{*},D_{y}^{*}=\underset{G_{x},G_{y},A_{x},A_{y}}{\arg\min}\left(\underset{D_{x},D_{y}}{\arg\max}\left(L\left(G_{x},G_{y},A_{x},A_{y},D_{x},D_{y}\right)\right)\right).$$

Finally, we can obtain the optimal parameters of $\alpha_{cyc}$ and $\beta_{Perceptual}$ by solving the minimax optimization problem, as described in Equation (8). These parameters represent the optimal solution obtained through continuous testing and calculation.

## 4. Experiments and Results

In this section, the proposed model is compared with various generative adversarial network models, including CycleDehaze [9], pix2pix [20], and the method of Mejjati et al. [26]. In addition, the proposed model is compared with some traditional methods, such as those presented by He et al. [3], Berman et al. [12], and Fattal [11]. We used the peak signal-to-noise ratio (PSNR), structural similarity (SSIM) [28], and CIEDE2000 [29] indicators to evaluate the models.

### 4.1. Dataset

Most current datasets use synthetic hazy images [30]. However, the proposed method uses the O-HAZE [10] and I-HAZE [31] datasets, which consist of 45 outdoor hazy images and 35 indoor hazy images, with corresponding haze-free images for each scene. Each image has a high spatial resolution, and the lighting parameters of the hazy and haze-free images are the same. The hazy images in this dataset were taken in actual hazy conditions generated using a professional haze machine. Unlike synthetic images, actual hazy images have different haze concentrations due to their depth and area. Thus, training and testing using this dataset provides a better reflection of the dehazing effect in a network model applying actual scenarios.

### 4.2. Implementation

Taking the O-HAZE [10] dataset as an example, during the training and testing phase, we used the TensorFlow [32] framework and mini-batch size to train our model with an NVIDIA GTX 2080Ti graphics card. The proposed network was trained using the produced training set including 9000 cropped images, before testing with the O-HAZE [10] dataset.

Due to the small number of images in the dataset, the proposed method enhanced the dataset by sequentially cropping the 45 original hazy images. To ensure the robustness of the dataset to different scales and textures, the position of the crop origin and the size of the crop were randomly generated. The cropped area was always a square, with length ranging from 256 pixels to the value of the smaller side of the image. If the distance between the crop origin and the image boundary was smaller than the length of the crop area, the image was discarded. To ensure that each image had the same weight in the dataset, the proposed method cropped each image to obtain 200 subimages, which were then scaled to a size of 256 × 256 pixels. The same method was used to randomly crop the haze-free images to form an unpaired dataset.

### 4.3. Results

The results of the experiment were compared with six other methods using the O-HAZE dataset [10]. Although the original intention of Mejjati et al.’s method [26] did not involve holistic changes, the author showed that the network can pay attention to the entire image and perform holistic changes when trained for enough epochs. In addition to the network structure, a further difference to that of the proposed method is that Mejjati et al. [26] used a trained attention map. We compared the difference between the attention map simulated using the dark-channel mechanism and the attention map trained using the method of Mejjati et al. [26]. We compared and tested the above methods in terms of both qualitative and quantitative aspects. Finally, we tested several additional sets of natural hazy images.

**Figure 4:**
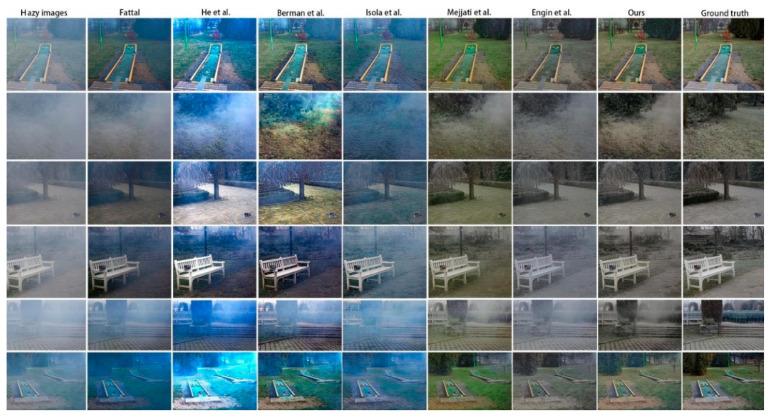
Dehazing results using the O-HAZE [10] dataset. The proposed method presented an improved dehazing effect and color reduction compared to other methods [3,9,11,12,20,26].

**Figure 5:**
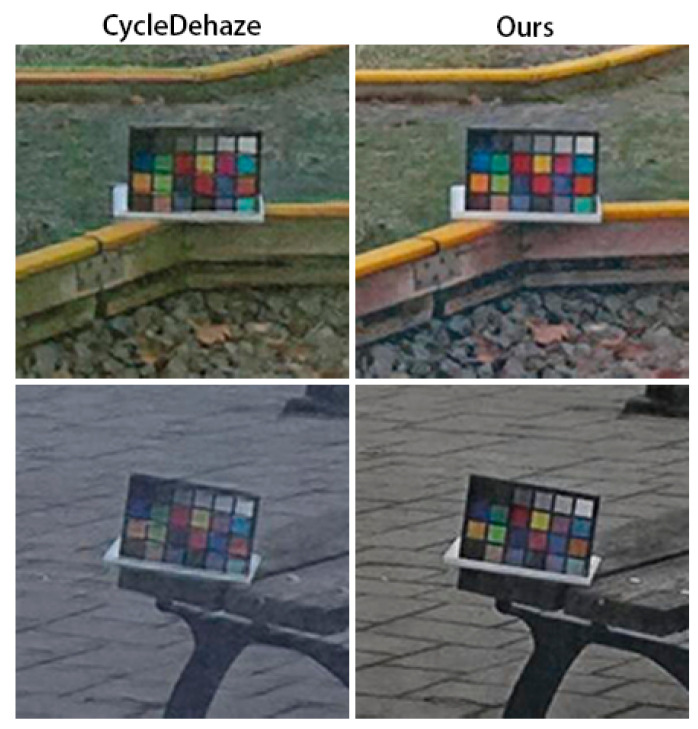
Comparison of the results generated using the proposed method and CycleDehaze [9].

Figure 4 shows a visual comparison of the results of several dehazing methods. The traditional methods of He et al. [3], Berman et al. [12], and Fattal [11] presented more serious color deviations. Many hazy areas were poorly removed by pix2pix [20], while the results of our model were closer to the ground truth. In addition, as shown in Figure 5, when magnified, the results of our model retained the features of the original image better than CycleDehaze [9], along with higher image clarity.

Table 1 presents the results of the proposed network’s PSNR, SSIM [28], and CIEDE2000 [29] indicators. PSNR and SSIM [28] are mainly used as image quality evaluation indicators, while CIEDE2000 [29] is usually used for color difference evaluation.

The peak signal-to-noise ratio (PSNR) [28] is the ratio of the energy of the peak signal to the average energy of the noise; it is one of the most widely used objective evaluation indicators for image quality. Since the mean square error (MSE) is the energy mean of the difference between the actual image and the noisy image, and the difference between the two images is the noise, the PSNR [28] indicator is then the ratio of the peak signal energy to the MSE. Equations (9) and (10) show the above-described calculation process.

(9) $$MSE=\frac{1}{N}\overset{N}{\underset{i=1}{\sum }}{\left(I\left(i\right)-\hat{I}\left(i\right)\right)}^{2},$$

(10) $$PSNR=10\;\log_{10}\left(\frac{L^{2}}{MSE}\right),$$

where MSE represents the mean square error between the actual image and the generated image, N represents the number of pixels in the image, and L represents the maximum pixel value. This indicator can reflect the average pixel error of two images.

The structural similarity (SSIM) [28] considers the similarity in terms of brightness, contrast, and correlation between two images. The calculation process uses the mean and variance of both images. Equations (11)–(14) show the above-described calculation process.

(11) $$SSIM={\left(l\left(x,y\right)\right)}^{\alpha }\;{\left(c\left(x,y\right)\right)}^{\beta }\;{\left(s\left(x,y\right)\right)}^{\gamma },$$

(12) $$l\left(x,y\right)=\frac{2\mu_{x}\mu_{y}+C_{1}}{\mu_{x}^{2}+\mu_{y}^{2}+C_{1}},$$

(13) $$c\left(x,y\right)=\frac{2\sigma_{x}\sigma_{y}+C_{2}}{\sigma_{x}^{2}+\sigma_{y}^{2}+C_{2}},$$

(14) $$s\left(x,y\right)=\frac{\sigma_{xy}+C_{3}}{\sigma_{x}^{2}\sigma_{y}^{2}+C_{3}},$$

where $C_{1}$, $C_{2}$, and $C_{3}$ are structural constants. In order to simplify the calculation, it is usually assumed that α=β=γ=1, $C_{3}=\frac{C_{2}}{2}$. Then, Equation (11) can be rewritten as follows:

(15) $$SSIM=\frac{\left(2\mu_{x}\mu_{y}+C_{1}\right)\left(2\sigma_{xy}+C_{2}\right)}{\left(\mu_{x}^{2}+\mu_{y}^{2}+C_{1}\right)\left(\sigma_{x}^{2}+\sigma_{y}^{2}+C_{2}\right)},$$

where $\mu_{x}$ and $\mu_{y}$ represent the mean value of both images, while $\sigma_{x}$ and $\sigma_{y}$ represent the variance of both images. A larger value of SSIM [28] denotes greater similarity between two images.

The CIEDE2000 [29] color difference formula was created by the International Commission on Illumination(CIE) in 2000 to improve the visual consistency of industrial color difference evaluation methods in distinguishing medium and small color differences in the surface of industrial products. Equation (16) shows its simplified calculation process.

(16) $$\Delta E_{00}^{*}=\sqrt{{\left(\frac{\Delta L^{*}}{K_{L}S_{L}}\right)}^{2}+{\left(\frac{\Delta C^{*}}{K_{C}S_{C}}\right)}^{2}+{\left(\frac{\Delta H^{*}}{K_{H}S_{H}}\right)}^{2}+R_{T}\left(\frac{\Delta C^{*}}{K_{C}S_{C}}\right)\left(\frac{\Delta H^{*}}{K_{H}S_{H}}\right)},$$

where $K_{L}$, $K_{C}$, and $K_{H}$ are coefficients adjusting the relatively wide capacity of brightness, saturation, and hue, and $R_{T}$ is a rotation function.

According to Table 1, it is obvious that the above-described indicators were superior when using the proposed method compared to CycleDehaze [9] in terms of detail and color, displaying higher values of PSNR and SSIM and a lower value of CIEDE2000 [28,29]. This shows that adding the attention mechanism improved the dehazing effect of the model and the quality of the generated images.

In order to prove the advantages of the proposed method, we performed a statistical test on the experimental results in Table 1. We used the Friedman Test [33] to sort and calculate the indicators of the 45 images used in the test, taking the indicators of the 45 images used in the test as the sample data for the hypothesis test, and sorting and calculating the results of each image in each comparison method. Finally, we calculated the p value for the chi-square statistic to be 1.14 × 10^−112^, the value of p reflects the probability that different algorithms have the same performance. Obviously, our p value is small enough to prove that the contrast method has a significant difference.

Then, we performed post-hoc test to further prove that the proposed method is significantly different from the method of Mejjati et al. [26] and Engin et al. [9]. We used the Nemenyi Test to calculate the critical value range CD=0.775. The difference between the average ordinal value of our method and the method of Mejjati et al. [26] is 1.282, and the average ordinal value of the method of Engin et al. [9] is 1.563. Both of these values are greater than CD; this proves that the proposed method has a significant improvement over these two comparison methods.

**Figure 6:**
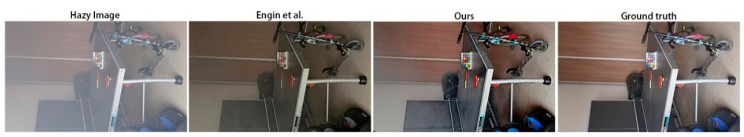
Dehazing results following the application of the proposed method and CycleDehaze [9] to the O-HAZE dataset [10].

We also compared the performance of the proposed method and CycleDehaze [9] using the I-HAZE dataset [31]. Figure 6 and Table 2 show some qualitative and quantitative results. It can be seen that our method was superior to CycleDehaze [9] when using the I-HAZE dataset [31].

In order to test the effect of our method across datasets, we conducted crossover experiments using models trained on the I-HAZE [31] and O-HAZE [10] datasets, followed by measuring the three indicators, with a subsequent comparison to CycleDehaze [9]. Table 3 shows the quantitative results of the cross-dataset evaluation, where it can be seen that the proposed model was again superior to CycleDehaze [9], thus proving our method’s generalizability.

**Figure 7:**
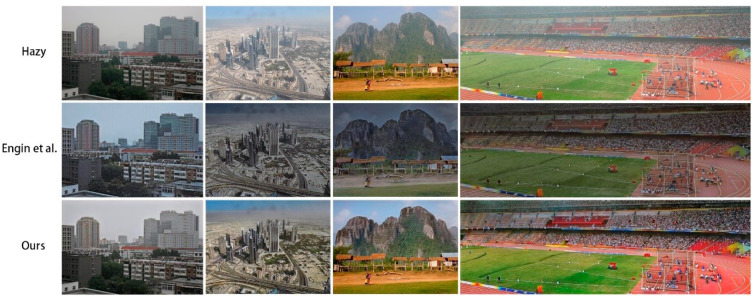
Qualitative results using natural hazy images in comparison with those generated by CycleDehaze [9].

In addition, Figure 7 shows the dehazing results of the collected actual hazy images. It can be seen that CycleDehaze [9] is darker in color and more blurry in detail at the edges of the object. In comparison, the proposed model removes haze more effectively, thereby obtaining better results.

**Figure 8:**
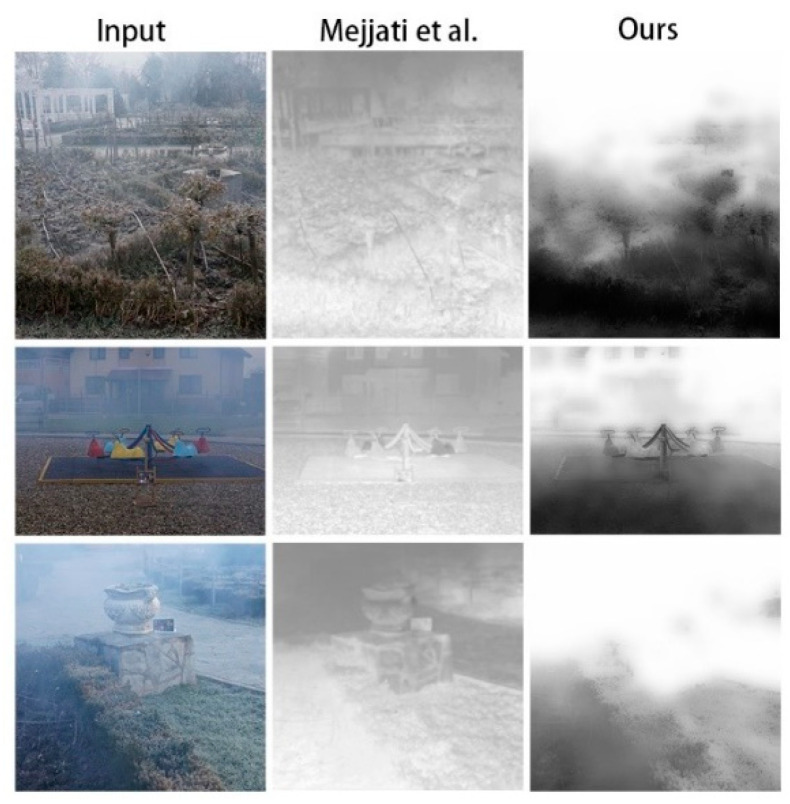
Results obtained with proposed attention map and that using the method of Mejjati et al. [26] through training of the network.

We also compared the results using different attention maps applied to hazy areas, mainly focusing on two types of attention maps: one obtained via self-training of the network and the other obtained via calculation using the dark channel. Mejjati et al.’s [26] model was used to obtain the first type of attention map, as shown in Figure 8. It can be seen that the attention map obtained via self-training does not adequately reflect the hazy area or its concentration, which may be related to the model algorithm. On the other hand, the proposed method enhanced the dark channel to obtain the second type of map, which allowed better indicating the hazy areas and their concentration in the image. Through the introduction of the enhancement coefficient *v*, an enhanced dark-channel attention map was obtained, as shown in the figure after training. It can be seen that the improved dark-channel attention map better shows the distribution and concentration of hazy areas than the attention map trained using the method of Mejjati et al. [26].

### 4.4. Dehazing Robustness

**Figure 9:**
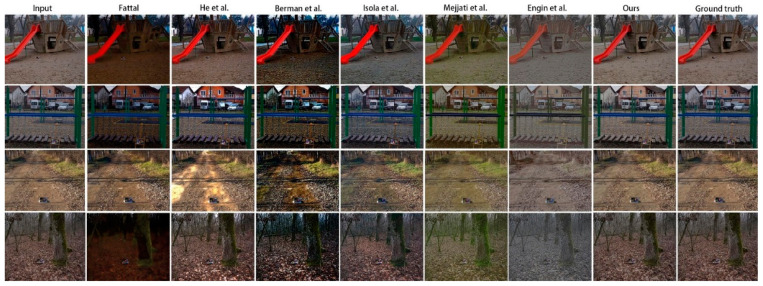
Translation results using our method and other methods for haze-free images [3,9,11,12,20,26].

**Figure 10:**
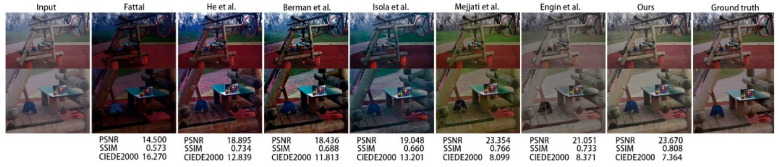
Comparison of the dehazing effects when applied to misty images [3,9,11,12,20,26].

To verify that the model could achieve the expected dehazing effect in any situation, we verified that the image was completely haze-free in extreme cases, as shown in Figure 9, and we verified the dehazing effect when applied to misty images, as shown in Figure 10. CycleDehaze [9] still converted the original clear images, resulting in color changes. Other methods such as dark channel also caused a large change in the generated images, which should not have occurred. Figure 10 shows the indicators describing the dehazing effect of each model when applied to the misty image. The proposed model was superior to other methods in terms of each quantitative indicator, especially color reduction. Additionally, we found that the method of Mejjati et al. [26], which also uses the attention mechanism, could restore the color and detail of the images relatively well in this experiment, showing that the attention mechanism has a significant role in dehazing haze-free and misty images.

In summary, as a result of the introduction of the attention mechanism, the proposed method can better approach this issue such that changes in the area are hardly noticeable; thus, there is no need to distinguish the haziness of the input image, thereby improving the robustness of the model.

### 4.5. Result Analysis

We compared the results obtained using the proposed method and others in the literature on the same dataset, where it was found that the proposed method’s sharpness, dehazing effect, and visual effect were better than those of other methods. When dehazing the misty and haze-free image areas, due to the introduction of the attention mechanism, the dark channels in the areas that were haze-free or that had little haze were closer to gray and black; thus, the value of each pixel in the attention map was biased toward 0. On the other hand, for the reserved area of the original image with no need for conversion, its weight was biased toward 1; thus, the characteristics of the original image could be better preserved. As shown in Figure 10, it can be clearly seen that our method could better retain the detail and color of the original image, thereby better restoring its original appearance.

## 5. Conclusions

The proposed method innovatively uses an enhanced dark-channel graph as the attention map added to CycleGAN [8]. This allows effectively improving the dehazing effect and sharpness of images from the same dataset, better preserving the detail of these images, and better restoring the original detail and color of misty and haze-free images. However, it is worth noting that the dark channel is still erroneous for areas marked with haze. The value of white objects in the dark channel is also greater; however, the area where these objects are located may not be hazy. Ideally, the attention map of a haze-free image should be completely black, but the proposed attention map cannot achieve this effect. Therefore, a method to accurately obtain hazy areas and their concentration, as well as improve the dehazing effect of the model, will be the focus of our future research.

## Figures and Tables

**Table 1:** Evaluation of quantitative indicators. This table lists the average peak signal-to-noise ratio (PSNR), structural similarity (SSIM), and CIEDE2000 [28,29] index results of 45 images from the O-HAZE [10] dataset using different models and methods. Our results are superior to those of other methods on the basis of these indicators.

	Fattal [11]	He et al. [3]	Berman et al. [12]	Isola et al. [20]	Mejjati et al. [26]	Engin et al. [9]	Proposed Method
PSNR ↑	15.502	13.267	15.235	14.371	18.430	18.664	18.732
SSIM ↑	0.577	0.631	0.620	0.555	0.645	0.595	0.674
CIEDE2000 ↓	17.710	23.505	18.073	20.790	11.808	11.175	10.955

**Table 2:** Evaluation of quantitative indicators. This table lists the average PSNR, SSIM, and CIEDE2000 [28,29] index results of 35 images from the I-HAZE [31] dataset.

	Engin et al. [9]	Proposed Method
PSNR ↑	16.428	17.125
SSIM ↑	0.768	0.783
CIEDE2000 ↓	13.072	11.965

**Table 3:** Cross-dataset quantitative results using the I-HAZE [31] and O-HAZE [10] datasets.

	Engin et al. [9]		Proposed Method	
	Training Set: I-HAZE Test Set: O-HAZE	Training Set: O-HAZE Test Set: I-HAZE	Training Set: I-HAZE Test Set: O-HAZE	Training Set: O-HAZE Test Set: I-HAZE
PSNR ↑	14.721	15.754	16.791	17.502
SSIM ↑	0.528	0.692	0.621	0.753
CIEDE2000 ↓	15.706	14.568	13.861	11.416
